# A randomized, parallel group, pragmatic comparative-effectiveness trial comparing medication-assisted treatment induction methods in primary care practices: The HOMER study protocol

**DOI:** 10.1371/journal.pone.0290388

**Published:** 2023-09-08

**Authors:** Douglas H. Fernald, Donald E. Nease, John M. Westfall, Bethany M. Kwan, L. Miriam Dickinson, Ben Sofie, Cory Lutgen, Jennifer K. Carroll, David Wolff, Lori Heeren, Maret Felzien, Linda Zittleman

**Affiliations:** 1 Department of Family Medicine, University of Colorado School of Medicine, Aurora, Colorado, United States of America; 2 Department of Family Medicine, University of Colorado School of Medicine (retired), Aurora, Colorado, United States of America; 3 Department of Emergency Medicine, University of Colorado School of Medicine, Aurora, Colorado, United States of America; 4 American Academy of Family Physicians, National Research Network, Leawood, Kansas, United States of America; 5 HOMER Community Advisory Council, Aurora, Colorado, United States of America; Yale School of Medicine, UNITED STATES

## Abstract

Opioid use disorder (OUD) represents a public health crisis in the United States. Medication for opioid use disorder (MOUD) with buprenorphine in primary care is a proven OUD treatment strategy. MOUD induction is when patients begin withdrawal and receive the first doses of buprenorphine. Differences between induction methods might influence short-term stabilization, long-term maintenance, and quality of life. This paper describes the protocol for a study designed to: (1) compare short-term stabilization and long-term maintenance treatment engagement in MOUD in patients receiving office, home, or telehealth induction and (2) identify clinically-relevant practice and patient characteristics associated with successful long-term treatment. The study design is a randomized, parallel group, pragmatic comparative effectiveness trial of three care models of MOUD induction in 100 primary care practices in the United States. Eligible patients are at least 16 years old, have been identified by their clinician as having opioid dependence and would benefit from MOUD. Patients will be randomized to one of three induction comparators: office, home, or telehealth induction. Primary outcomes are buprenorphine medication-taking and illicit opioid use at 30, 90, and 270 days post-induction. Secondary outcomes include quality of life and potential mediators of treatment maintenance (intentions, planning, automaticity). Potential moderators include social determinants of health, substance use history and appeal, and executive function. An intent to treat analysis will assess effects of the interventions on long-term treatment, using general/generalized linear mixed models, adjusted for covariates, for the outcomes analysis. Analysis includes practice- and patient-level random effects for hierarchical/longitudinal data. No large-scale, randomized comparative effectiveness research has compared home induction to office or telehealth MOUD induction on long-term outcomes for patients with OUD seen in primary care settings. The results of this study will offer primary care providers evidence and guidance in selecting the most beneficial induction method(s) for specific patients.

## Introduction

Opioid use disorder (OUD) leading to major morbidity and overdose death is a major health issue in the United States [1, 2]. As the prevalence of OUD has increased to epidemic proportions, so has the number of overdose deaths attributed to opioids [3, 4]. Estimated drug overdose deaths increased nearly 30% between May 2020 and April 2021, with 75% of overdoses involving opioids [5].

Office-based opioid treatment is the ambulatory care provision of medication for opioid use disorder (MOUD) for patients suffering from opioid use disorder. MOUD with buprenorphine is a safe and effective strategy to treat OUD and reduce overdose deaths [6]. MOUD is accessible to patients through primary care [3, 4, 7, 8]. Treating patients with buprenorphine involves an initial induction, during which patients discontinue opioids, begin withdrawal, and receive the first few doses of buprenorphine. National guidelines for MOUD have focused on office induction (synchronous, observed in-person) to begin buprenorphine [3]. Timing the last use of opioids such that patients arrive during available office hours for induction while in withdrawal is challenging. Further, office induction is a resource intensive activity, and some practices are reluctant to dedicate this amount of resource for one patient encounter [9, 10]. Although resource intense, these same office resources might also create a positive cumulative benefit for patients not available in home inductions, including enhanced patient-provider-practice interaction and support for patient behavioral factors (motivation, planning, and action control) known to affect long-term maintenance treatment [11–14].

An alternative is home MOUD induction, which also offers a safe, guideline concordant approach to care [3, 4]. For home induction, patients can stop opioids at any time and start buprenorphine when they reach the appropriate level of withdrawal. Stigma associated with OUD and difficulty with transportation may present other barriers to starting MOUD at the clinic [9, 15, 16]. Some patients may prefer undergoing MOUD induction in the relative comfort of their own home. However, while potentially more convenient, some patients lack social supports and relationships [17–19] and the cognitive ability to set goals and follow through on plans (low executive function) often necessary for successful MOUD induction and treatment [20–22].

A third MOUD induction approach using telehealth emerged during the COVID-19 pandemic, allowing patients access to induction via synchronous, observed phone or video contact with practice staff. Comparative effectiveness of office, home, and telehealth MOUD induction for short-term stabilization and long-term maintenance treatment is not known.

Distinctions among office, home, and telehealth-based MOUD induction processes and patient experience may influence short-term stabilization (30 days from induction initiation) and long-term maintenance (60, 90, and 270 days from induction initiation). As a chronic disease, long-term adherence to buprenorphine for OUD is critical for reduction of overdose deaths [23]. MOUD often involves intermittent return to opioid use and treatment lapses, requiring multiple attempts at long-term recovery. The clinical and social context in which initial induction occurs (e.g., with support from clinical staff in the office or via telehealth or independently or with supportive care partners in the home) may influence patient motivation and planning for early phase treatment adherence. Early phase treatment adherence (within the first 30 days of induction) may subsequently influence development of habits and routines needed for long-term adherence.

No large-scale, multi-center randomized comparative effectiveness research has compared office, home, and telehealth induction on long-term outcomes for patients with OUD seen in primary care. There is currently insufficient evidence to favor and recommend home-, office-, or telehealth-based induction either globally or based on individual patient characteristics [3, 4, 7]. The purpose of this paper is to report the research protocol for the “Home vs Office vs Telehealth for Medication Enhanced Recovery” or HOMER study. Results from this study will help to clarify recommendations for induction methods for patients with OUD.

### Objectives

The objectives of this study are to: (1) compare short-term stabilization and long-term maintenance treatment in MOUD in patients receiving office induction (synchronous, observed), home induction (asynchronous, unobserved), or telehealth induction (synchronous phone or video contact, observed), using multiple two-way comparison; and (2) identify clinically relevant practice and patient characteristics associated with successful long-term treatment.

### Hypotheses

This study will examine the following hypotheses:

1. The number of days of taking buprenorphine will be different among patients using office, home, or telehealth induction at 30-, 90-, and 270-day follow-up.
2. The percent of patients who take buprenorphine on ≥80% of the days between day 1 and day 270 will be different among patients using office, home, or telehealth induction at 30-, 90-, and 270-day follow-up.
3. Improvements in patient-reported quality of life and potential mediators of maintenance treatment (intentions, planning, and automaticity) over a 270-day follow-up period will be different among patients using home induction, office induction, or telehealth induction at 270-day follow up.
4. Social determinants of health, substance use history and appeal, and executive function will moderate effects of induction methods on outcomes. Thus, we predict that executive function will moderate comparative effectiveness of office‐based versus home MAT induction. Patients with high executive function, shorter history of substance use, and lower substance use appeal may be more successful with home induction. Patients with lower executive function, longer history of substance use, and greater substance use appeal may be more successful with office‐based MAT induction.”

**Fig 1:**
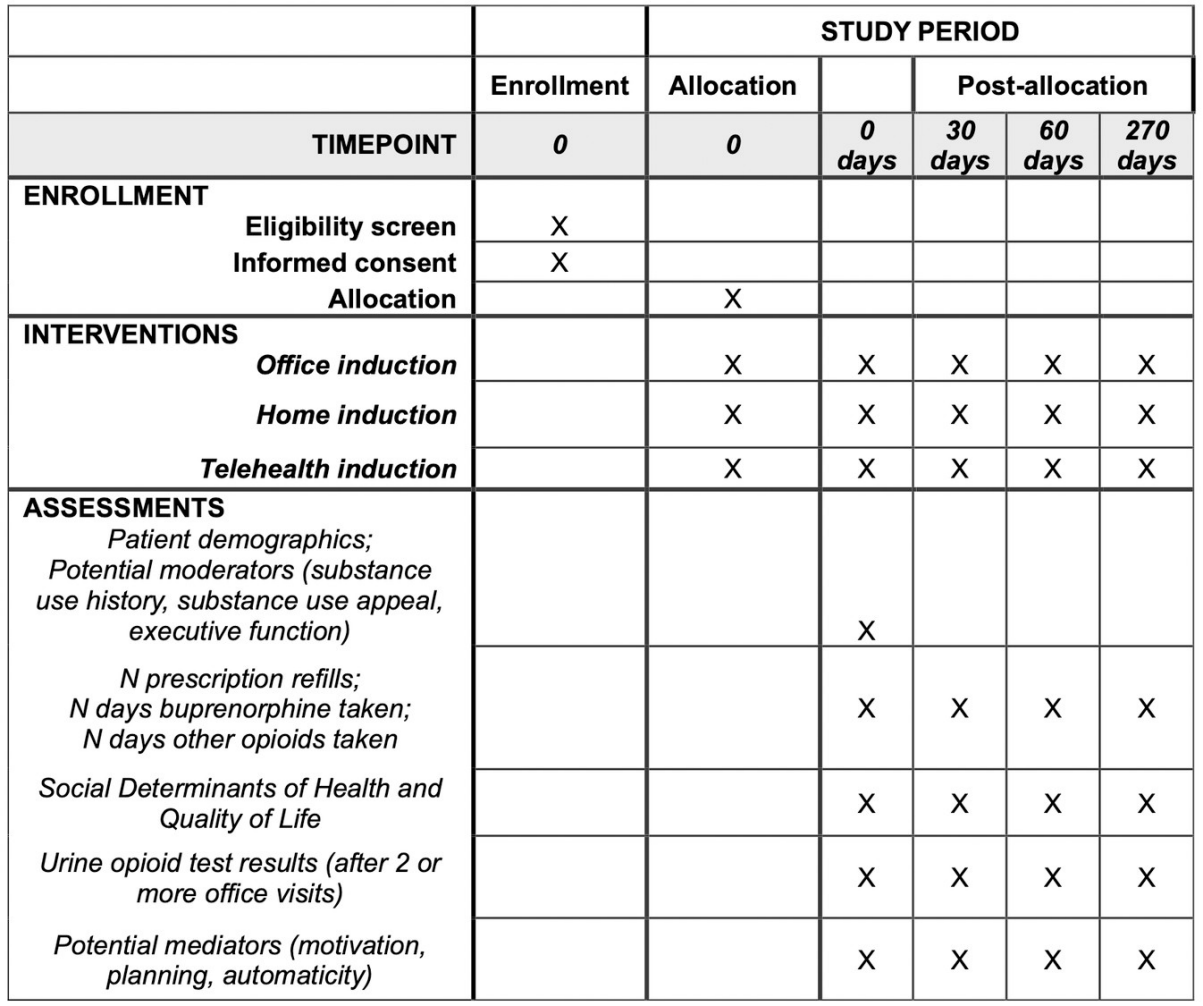
Schedule of enrollment, interventions, and assessments.

Patient-level data collection begins at the time of randomization to one of the three MOUD induction method intervention arms (Fig 1).

## Materials and methods

### Conceptual framework

**Fig 2:**
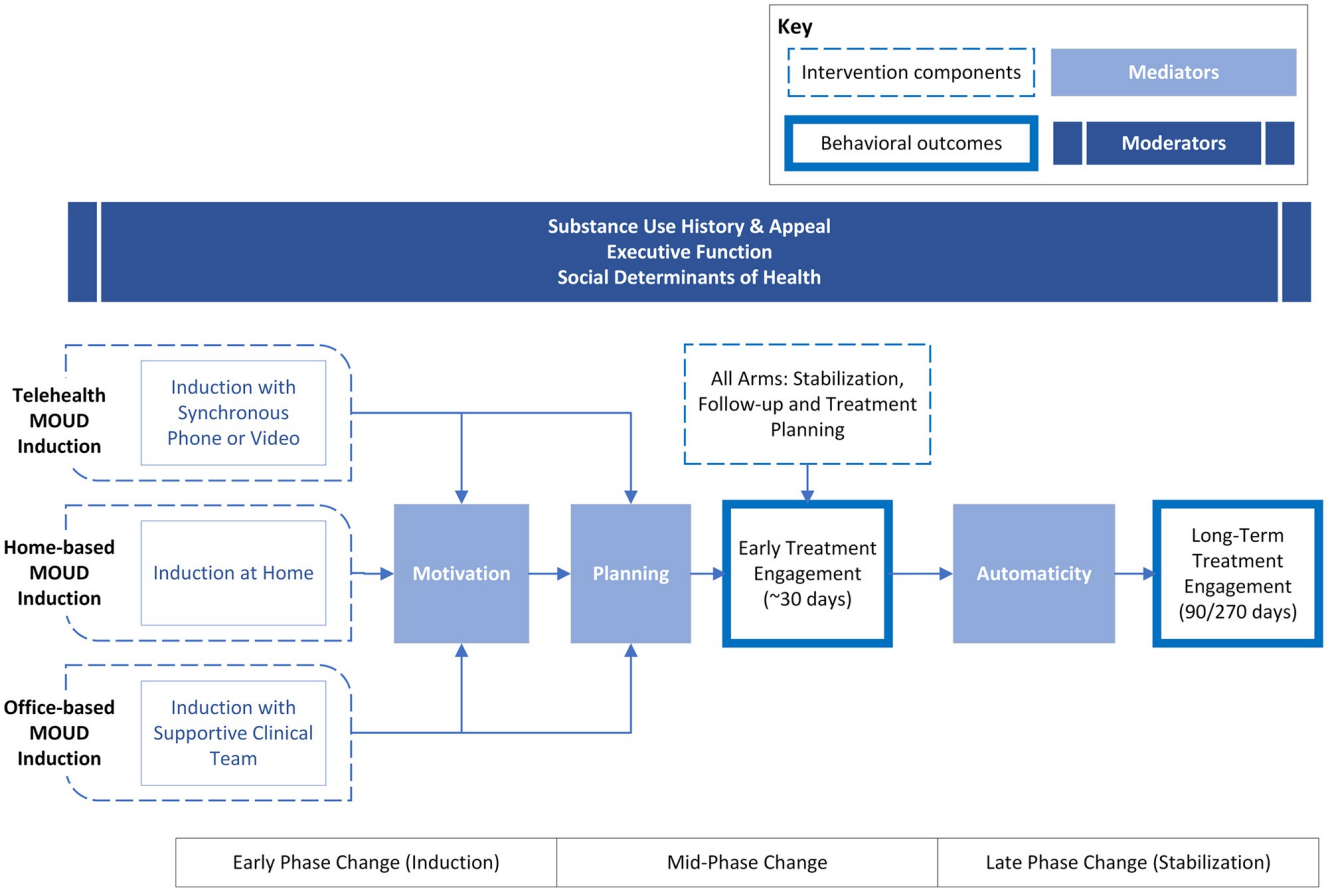
Conceptual model for effects of three methods of medication for opioid use disorder (MOUD) induction on long-term maintenance treatment.

The HOMER Study conceptual framework (Fig 2) is guided by concepts from the Rubicon model of action phases [24] and self-determination theory applied to health contexts [25]. Medication-taking (behavioral outcome) is a function of self-determined motivation and planning (early phase mediators; predictors of treatment at 30 days) and automaticity (late phase mediator; predictor of maintenance treatment at 90 and 270 days). Potential patient-level moderators of effects of the intervention on planning and medication taking include social determinants of health, executive function, history of substance use, and substance use appeal.

### Design and setting

This study uses a randomized, parallel group, three-arm pragmatic comparative effectiveness trial design of three standard of care models for MOUD induction in primary care. Randomization occurs at the patient level. The setting is primary care practices in the United States with at least one prescriber currently treating patients with OUD with buprenorphine. Practice recruitment was done through the State Networks of Colorado Ambulatory Practices and Partners (SNOCAP) or the American Academy of Family Physicians National Research Network (NRN) practice-based research networks and most participating practices are affiliated with these networks.

### Practice enrollment

Practices will be recruited through the SNOCAP and NRN practice-based research networks. Information about the study is disseminated through professional collaborations and individual relationships of study team members. Practices receive an email that includes two-page an informational recruitment flyer. Study personnel conduct a 30-minute “Overview Call” with interested practices to describe study goals and practice activities, expectations, and compensation. Practices receive $200 for every patient enrolled and $35 per patient per month for follow-up clinical data.

Practices receive patient informational packets along with posters and flyers to display at the practice. Study support is provided to practices through monitored email and a toll-free hotline.

### Study population

Patients whose primary care provider in a participating practice believes they would benefit from MOUD with buprenorphine and who agree to be randomized are eligible. Patient participants are aged 16 years and older who are identified by their clinician as having opioid dependence and either: 1) substance use disorder as defined by DSM-V criteria for OUD; or 2) chronic pain with long-term, high dose opioid use (greater than one year and morphine equivalent daily dose higher than recommended by the Centers for Disease Control and Prevention); or both. Patients will be excluded if they are hypersensitive to buprenorphine or naloxone, known to have serum aspartate aminotransferase or alanine aminotransferase levels greater than five times normal, and/or diagnosed with severe, untreated psychiatric illness. If a patient is deemed ineligible for study participation or does not wish to participate, MOUD induction will occur using the practice’s usual clinical protocol.

### Sample size

The study plan is to recruit 100 practices and enroll 1400 patients to reach a goal of 1200 patients receiving MOUD induction, allowing for attrition prior to MOUD induction. For some outcomes (e.g., quality of life), we allow for ~20% attrition after receiving MOUD induction (leaving 960 patients total, 320 per arm). With patient-level randomization, the impact of clustering within practices is minimal and unlikely to impact conclusions. For the dichotomous outcome of maintenance treatment, a sample size of 320 patients per arm will provide 80% power to detect a 12% difference in long-term maintenance treatment between study arms with alpha = .05 (e.g., 45% vs 57%). For continuous outcomes (e.g., quality of life, % of days taking buprenorphine out of 270 days), a sample size of 320 patients per arm will provide 81% power to detect a .23 SD difference (small) between any two study arms with alpha = .05.

### Patient enrollment, consent, and allocation

Clinicians and staff at participating primary care practices identify and refer interested and potentially eligible patients who are starting MOUD to the central study team. Patients starting or considering MOUD at a participating practice can also directly contact the study team if they receive information about the study at the practice by calling the toll-free hotline, staffed during extended business hours (to accommodate multiple time zones) Monday through Friday. A study team member calls patients, describes the study purpose, verifies eligibility, conducts informed consent, and randomizes patients who agree to participate.

Consented participants are randomized to the office, home, or telehealth MOUD induction arm using block randomization to ensure approximately equal numbers of patients in each arm. Directly after randomization, the study team member informs the participant of their randomization assignment. Immediately following randomization, an automated message is emailed to designated practice members with the randomization result and the study team member calls the practice with the result.

Data on practice characteristics and moderators (Practice Culture Assessment, [26] Practice Characteristics Survey, and MAT Implementation Checklist [27]) are collected at the time of practice enrollment only.

### Comparator interventions

Induction interventions may be performed using one of the three comparator induction protocols: office, home, or telehealth. All three induction methods are evidence-based and considered standard care [16, 28–30]. For all induction methods, MOUD with buprenorphine begins with induction, during which patients discontinue their opioids, begin withdrawal, and receive the first one to four doses of buprenorphine. The neurobiology of opioid tolerance requires that a person achieve a level of partial withdrawal for buprenorphine initiation to not result in a precipitated withdrawal effect [6, 31]. This requires patients take their last opioids 24–72 hours prior to induction. If given too early, buprenorphine can rapidly precipitate an intense opioid withdrawal. Regardless of the induction method, all study participants remain in the care of their primary care provider who will continue to manage and oversee the care of enrolled patients.

#### Comparator intervention 1: Office induction

For office induction, a patient receives instruction on induction process from the clinic team at an in-person or telehealth visit. On a pre-determined day, the patient stops taking opioids and comes to the office with mild to moderate withdrawal. The clinic team monitors the patient, assesses symptoms using the Clinical Opiate Withdrawal Scale (COWS) to determine time of first dose of medication, and administers first dose with the patient [32]. The office induction includes the observed administration of the first dose, followed by in-person observation and evaluation 30–60 minutes after the first dose. At this time (at least 30–60 minutes after first dose), the clinic team and patient decide whether the second dose should be administered in the office or if the patient is able to leave the clinic to self-administer subsequent doses.

#### Comparator intervention 2: Home induction

Home induction is done primarily by the patient in their current residence. The patient receives instruction on the induction process from their clinic team at an in-person or telehealth visit. Home induction is initiated by the patient at a time and place (other than the clinical office) determined by the patient. The patient determines when to stop taking opioids, begins withdrawal, monitors symptoms, administers the Subjective Opiate Withdrawal Scale (SOWS), and determines when to take first dose of medication, per the instructions and protocol provided [33]. The clinic team does not observe or have contact with the patient while the patient undergoes these steps or takes the first dose. The patient continues this process for additional doses. Follow-up contact with clinic team may occur after the first or second day, typically within a week.

#### Comparator 3: Telehealth induction

For telehealth induction, a patient receives instruction on induction process from the clinic team at an in-person or telehealth visit. The patient undergoes the same process as an office induction but from a location other than the clinic. Like an office induction, the patient has regular contact with someone from the practice team on Day 1 of induction. Prior to initiating the first dose, the patient has contact by phone or video with the clinic team to assess symptoms and determine the level of withdrawal (using COWS or SOWS). The administration of the first dose of medication is determined by the clinic team during phone or video contact, and the clinic team is in contact with the patient by phone or video when the first dose is taken. This process continues through the second and possible third dose. The patient is re-assessed via video or phone regularly by clinic staff and prescriber throughout this process.

### Study assessments and measures

#### Primary patient outcomes

The primary patient-level outcomes are (Table 1):

1. Number of days buprenorphine taken between day one and day 270 of treatment
2. Number of days illicit opioids taken between day one and day 270 of treatment.
3. Percent of patients in each group who took buprenorphine on ≥80% of the days between day one and day 270.
4. Percent of patients in each group taking illicit opioids on <10% days from induction through 270-day follow-up.

**Table 1:** Patient and assessments and measures.

Instrument	# of items	Timing (days from baseline)			
		0	30	90	270
**PATIENT MEASURES AND ASSESSMENTS**					
**Primary outcome measures**					
Number of days buprenorphine taken [34]	1		X	X	X
Number of days other opioids taken	1		X	X	X
**Moderators**					
PROMIS Appeal of Substance Use (Past 3 months) v1.0 –Short Form 7a [35]	8	X			
PROMIS Severity of Substance Use (Past 3 months) v1.0 –Short Form 7a [35]	8	X			
Behavior Rating Inventory of Executive Function–Adult [36]	75	X			
AAFP Physicians Social Needs Screening Tool [37]	9	X	X	X	X
Everyday Discrimination [38]	5	X	X	X	X
UCLA 3-Item Loneliness Scale [39]	3	X	X	X	X
**Mediators**					
Health Care Climate Questionnaire [40, 41]	7	X	X	X	X
Action Planning and Coping Planning Assessment [13]	12	X	X	X	X
Self-Report Habit Index [42]	12		X	X	X
**Additional patient characteristics and co-variates**					
CDC Healthy Days Measures [43]	4	X	X	X	X
Generalized Anxiety Disorder 7-item scale [44]	7	X	X	X	X
Patient Health Questionnaire 9 [45, 46]	10	X	X	X	X
PEG: 3-Item Pain Intensity & Interference Scale [47]	3	X	X	X	X
Sense of Coherence Scale Short Form [48]	9	X	X	X	X
Perceived Stress Scale—Short [49]	2	X	X	X	X
Side Effects	1	X	X	X	X
**Demographics**					
Age, race/ethnicity, education, gender, marital status, household income, health insurance, military service	10	X			
**PRACTICE MEASURES AND ASSESSMENTS**					
Practice characteristics: region, practice type, university affiliation, residency site, size, patient population, telehealth use	9	X			
MAT Implementation Checklist [27]	21	X			
Practice Culture Assessment [26]	22	X			

#### Secondary patient outcomes

1. Quality of life and potential mediators (motivation, planning, and automaticity)

The standard of care is for patients to self-report the number of days they have taken buprenorphine, to provide samples for urine opioid tests for other opioids (including illicit opioids), and for practices to monitor buprenorphine prescriptions (initial and refills). The primary clinical outcome data—buprenorphine use, illicit opioid use, and other opioid use—will be recorded for each participating patient by practice staff using a structured data entry form. The mediators and quality of life outcomes data will be collected by patient survey completed at baseline, 30 days, 90 days, and 270 days.

#### Patient-level moderators and mediators

Patient-level moderator items will be collected by survey at baseline. Patient-level mediators will be collected by patient survey completed at baseline, 30 days, 90 days, and 270 days. Patients will have the option to complete surveys by telephone (interviewer-administered), on paper or online (self-administered).

#### Practice-level data

Practice measures will be collected at baseline using a practice characteristics survey to be completed by a knowledgeable person in each practice to capture details about practice type, number of providers, and patient population demographics. At baseline, all individual practice clinicians and staff will complete the Practice Culture Assessment [26] to capture details about practice change culture, work culture, and chaos. Practice process measures will be collected by survey at baseline from knowledgeable practice members who will complete the MAT Implementation Checklist [27] to assess MOUD treatment protocols.

### Quantitative data analysis

After generating initial descriptive statistics on patient and practice characteristics, analysis will evaluate baseline characteristics of patients in all three arms using chi-square tests for categorical variables and ANOVA for continuous variables to assess for unbalanced confounders. Patient-level covariates (to adjust for potential confounding or associated with loss to study follow-up for completion of surveys and/or practice follow up for documentation of continuation in treatment) and potential moderators may include age, gender, race/ethnicity, comorbidities, and other sociodemographic variables. In the event normality assumptions are not met, transformations will normalize distributions, ordinal or Poisson regression where appropriate, and/or the appropriate link function (e.g., logit link for dichotomized measures) [50–52].

Intent to treat (ITT) analyses will use general (generalized) linear mixed models to incorporate hierarchical and/or longitudinal data structures for primary and secondary outcomes [53–58]. All randomized patients for whom we have any data will be included in the ITT analysis, assigned to the group to which they were randomized, regardless of whether they received the assigned induction or any induction at all. Even though randomization will be at the patient level, patients are still clustered within practices. Therefore, analysis will include practice random effects as well as patient random effects (for longitudinal data). Likelihood based approaches using all available data, adjusting for covariates that are associated with missingness, are the preferred method for analyzing longitudinal data with missingness under missing at random conditions [59–61]. Hypothesis tests will be two-sided with alpha = .05 or p values reported. Goodness of fit statistics and model fitting diagnostics will be used to assess influential points, outliers, overdispersion and heteroscedasticity and to evaluate alternative model specifications. All statistical analyses will be performed using SAS version 9.4 (SAS Institute Inc., Cary, N.C.).

Additional exploratory analyses will test for other potential effect modification (moderator of intervention effectiveness) by selected patient characteristics as well as potential mediators of intervention effectiveness. The general hypothesis for these moderator analyses is as follows: The effects of the home versus office versus telehealth-based induction on patient medication-taking (and other primary outcomes) will differ by baseline levels of executive function, substance use (amount and shorter history), and social determinants of health, dichotomized by baseline scores.

### Qualitative data collection and analysis

We will conduct semi-structured practice staff and clinician interviews in up to 40 participating practices to capture the overall experience with HOMER and to collect important data about the implementation and sustainability of MOUD and the different induction methods. To select the subset of practices invited to participate in qualitative interviews, we will use a stratified maximum variation sampling method on based on final practice characteristics of enrolled practices [62]. The stratification method aims to purposefully select a wide range of practice types (e.g., size, patient population, number of waivered providers, time from baseline) while attempting to sample several practices within key strata (e.g., region, practice type). Such sampling will document diversity in specific implementation details such as staffing, workflows, experience with MOUD, and adaptations. Stratification will ensure there is sufficient data collected within to identify possible patterns within dimensions of interest. Interviews will take place in months 18–30 of the overall study timeline, once practices have reached the 270-day follow-up phase with patients, including practices that may not have successfully enrolled patients in the study.

Study staff will maintain monthly semi-structured field notes that document implementation data about the practice experience with patient enrollment and tracking, as well as other study implementation questions, concerns, and lessons.

Analysis of practice interview and field note data will be conducted by two qualitative researchers with ongoing input and direction from the principal and co-investigators. Interview data will be transcribed, cleaned, and entered in the ATLAS.ti qualitative software program along with field note data and organized by practice. For all analyses, we will begin with a grounded hermeneutic editing approach to help identify themes that are “grounded” or developed from an interpretation of the data [63]. Emergent themes will be shared with the rest of the study team for comments and questions before the analysts return to the data for further coding and review. Case-based matrices will be used iteratively with the study team to further organize the coding schemas into refined categories and to help identify common themes and patterns in the data, plus contradictory and unique cases or findings [64].

### Data management

All quantitative data (e.g., surveys, clinical data, tracking data, and participant management data) will be entered into and stored in a secure, structured database with field- and form-level validation controls to minimize data entry errors using REDCap, a HIPAA-compliant, web-based application for research data collection and management [65]. Responses completed by paper survey or telephone will be entered into the same REDCap database.

### Data safety and monitoring

Study staff will conduct ongoing monitoring of patient and practice enrollment and patient and practice data, including survey completion rates, missing data, and patient attrition. An independent Data and Safety Monitoring Board (DSMB) will convene to assess study progress, the safety data, and critical efficacy endpoints (as appropriate) and provide recommendations. The DSMB review cumulative study data to evaluate safety, study conduct, and scientific validity and data integrity of the study. The DSMB members include a biostatistician, a family physician researcher, a clinical pharmacist, a general internist and addiction medicine specialist, and a clinical psychologist. A patient’s primary care provider and care team continue to oversee and provide all usual patient care, including the management of any OUD treatments.

Random identifiers will be assigned to patients, which will be stored in the practices’ databases to allow linkage of clinical and survey data. All patient-level data will be stripped of direct identifiers before submission to the study team.

### Ethical considerations and declarations

This study has been reviewed and approved by the primary study site human subjects review board, Colorado Multiple Institutional Review Board (COMIRB). Participating practices covered by other review boards ceded human subjects oversight to COMIRB or completed their own review and approvals. Verbal consent is obtained for all study subjects. A waiver of documentation of consent was approved by the human subjects review board. Protocol modifications will be submitted for approval to the institutional review boards prior to implementation. This trial is registered at ClinicalTrials.gov (ID NCT04664062, last update: August 11, 2022). The principal investigators declare no financial conflicts or other competing interests.

### Trial status and timeline

This manuscript describes version 3/24/2022 of our protocol. Enrollment to the study began in October 2020. Due to disruptions from the COVID pandemic, the original patient enrollment period was extended, expecting to be completed by June 2023. Data collection for the primary outcomes will continue for approximately 270 days past last patient enrolled, expecting to be completed by April 2024.

## Discussion

### Summary

The HOMER study is a pragmatic comparative-effectiveness study [66]. Treatment with buprenorphine for opioid dependence and OUD in primary care is a crucial step toward addressing the epidemic overdose death rates. However, a significant gap in knowledge exists regarding the comparative effectiveness of office versus home versus telehealth induction for MOUD, despite acknowledged viability of home induction in current guidelines.

### Strengths and limitations

Answering questions related to MOUD induction will decrease barriers to implementing MOUD, expanding access to this life-saving treatment. Strengths of this study include its setting in primary care practice-based research networks, including safety net and rural practices, which generates pragmatic results from real-world settings. Findings will be immediately applicable to primary care settings and clinicians with a range of experience of MOUD. This study will include a substantially larger sample and longer follow-up period than previous research. The randomized study design increases our ability to more clearly understand which induction method for MOUD is better for which patients. Inclusion of the telehealth MOUD (in addition to office and home) induction method provides the best opportunity to evaluate all options for a customized, patient-centered approach in a COVID-19 world. Finally, this study is the direct result of questions and comments regarding office versus home inductions for MOUD from practices, patients, community members, stakeholders, and practice facilitators involved with the Implementing Technology and Medication Assisted Treatment Team Training in Rural Colorado (IT MATTTRs) Program [27]. Community and patient partners contributed to the development of this project proposal and study protocol, including consent process, timing of patient data collection, and practice eligibility criteria, and patient survey development, which enhances pragmatic execution of the trial.

A limitation of this study is that the study population includes people who may be experiencing transition and uncertainty in their life, so the risk for being lost-to-follow up may be higher than other study populations, impacting longitudinal data collection for some enrolled patients. Because this study is being done in more than 50 practice sites, we are not able to standardize the individual patient treatment. Dosing and other treatments including behavioral health treatment, job training, education are not included in our protocol. This study is also only relevant to MOUD induction methods for patients receiving care in primary care settings; results are not expected to generalize to patients being induced in emergency medicine or other specialty care settings. However, the strength of this study is that it takes place in primary care settings that are common throughout the community and the patients are those accessing primary care.

### Dissemination plans

Dissemination of research findings requires commitment to both peer reviewed presentation and publication and to strategic efforts with patient and community partners and key stakeholders. Throughout the course of this study, we will distill lessons learned and share findings throughout the life of the grant to local providers, practices, and communities. We will work closely with the University of Colorado and the American Academy of Family Physicians (AAFP) media departments. We will share findings through peer-reviewed publications and present our work efforts and findings at pertinent national primary care, substance use and addiction medicine, and public health meetings. We will also create template articles for practice members to share with their local news outlets over the course of the study.

### Conclusion

With opioid use and drug-related deaths increasing, study findings will be critical and timely. Answering questions related to MOUD induction will decrease barriers to implementing MOUD, expanding access to this life-saving treatment. The results of this study will help determine if there is an optimal induction approach to MOUD overall and for different patients based on measurable and clinically relevant factors. Results will help patients and providers make informed decisions related to MOUD, improve patient experience, and reduce morbidity and mortality due to opioid dependence and OUD.

## Supporting information

S1 Checklist SPIRIT checklist. (DOCX)

S1 File Study protocol. (PDF)
