# Correction: A randomized, parallel group, pragmatic comparative-effectiveness trial comparing medication-assisted treatment induction methods in primary care practices: The HOMER study protocol

**DOI:** 10.1371/journal.pone.0321303

**Published:** 2025-03-27

**Authors:** Douglas H. Fernald, Donald E. Nease, John M. Westfall, Bethany M. Kwan, L. Miriam Dickinson, Ben Sofie, Cory Lutgen, Jennifer K. Carroll, David Wolff, Lori Heeren, Maret Felzien, Linda Zittleman

The following information is missing from the funding statement: Research reported in this publication was funded through a Patient-Centered Outcomes Research Institute (PCORI) Award (IHS-2019C1-16167). The statements in this publication are solely the responsibility of the authors and do not necessarily represent the views of the Patient-Centered Outcomes Research Institute (PCORI), its Board of Governors or Methodology Committee.
