# Supplementary material for: A randomized, parallel group, pragmatic comparative-effectiveness trial comparing medication-assisted treatment induction methods in primary care practices: The HOMER study protocol
Source: PLoS One. 2023 Sep 8;18(9):e0290388. doi: 10.1371/journal.pone.0290388 (PMC10490863; doi:10.1371/journal.pone.0290388)
Supplement: S1 File — (PDF) [file pone.0290388.s002.pdf]

COMIRB Protocol

COLORADO MULTIPLE INSTITUTIONAL REVIEW BOARD
CAMPUS BOX F-490 TELEPHONE: 303-724-1055 Fax: 303-724-0990

Protocol #: 20-1692
Project Title: Comparing Office and Home Induction for Medication Assisted Treatment for Opioid Use Disorder (HOMER)
Principal Investigator: Linda K Zitttleman, MSPH
Version Date: 8-24-2020

I. Hypotheses and Specific Aims: A brief statement of the purpose of the research project. This section should include the hypotheses and specific aims being tested in the research. *Note: If you are applying for QA/QI/PE determination, you should not use terms like "hypothesis" or "research;" Instead, use the terms "aims" or "goals" and "evaluation."*(Approx. 1 paragraph)

The purpose of this study is to compare short-term stabilization and long-term maintenance treatment outcomes of home medication assisted treatment (MAT) induction to office MAT induction for patients suffering from opioid use disorder (OUD) and opioid dependence.

Specific Aims:

1. Compare short-term stabilization and long-term maintenance treatment engagement in MAT in patients receiving home induction to those receiving office-based induction.
2. Identify clinically relevant practice and patient characteristics associated with successful long-term treatment.

Hypotheses:

- H1. Patients using observed (office-based) MAT induction will have a greater number of days of buprenorphine treatment than patients using unobserved (home) MAT induction at nine-month follow-up.
- H2. Patients using office-based MAT induction will be more likely to take buprenorphine on ≥80% of the days between day 1 and day 270 than patients using home MAT induction.
- H3. Patients using office-based MAT induction will report greater improvement in quality of life, social determinants of health and social functioning over a nine-month follow-up period than patients using home induction.
- Hypotheses 1a, 2a, 3a: Intervention effects on primary outcomes (medication-taking and quality of life) will differ for patients in subpopulations of interest, defined by high executive function, shorter history of substance use, and lower substance use.

II. Background and Significance: Explain the background of this project so that we will understand why it is important to perform this research project. (Approx. 1 page)

The United States faces the worst drug overdose epidemic in its history.[1] Annual deaths from opioid drug overdose now exceed the number of people dying in car accidents.[2] A key driver of the overdose epidemic is a sharp increase in the prevalence of OUD.[3,4]

Office-based Opioid Treatment (OBOT) is the primary care or ambulatory care provision of medication assisted treatment (MAT) for patients suffering opioid use disorder (OUD). MAT with buprenorphine in primary care clinics is a proven strategy to treat opioid use disorder (OUD) and is slowly becoming accessible to patients through primary care.[5,6,7,8] Treating patients with buprenorphine involves an initial induction, during which patients discontinue their opioids, begin withdrawal, and receive the first few doses of buprenorphine. National guidelines for OBOT have focused on observed (office-based) induction to begin MAT.[9] Over the years, unobserved (home) MAT inductions have also been used and shown to be safe and effective. Individually, each induction strategy is evidence-based, guideline concordant care.[10,11] Most research on which the current guidelines are based examined short-term outcomes. However, OUD is a chronic condition. MAT often involves intermittent return to illicit opioid use and treatment lapses, resulting in multiple attempts to remain in long-term treatment. Important differences between the activities that occur during a home versus an office-based induction might influence short-term stabilization, long-term maintenance treatment, and quality of life outcomes. No large-scale, multi-center, randomized comparative effectiveness research has compared home versus office-based induction on long-term outcomes for patients suffering from OUD seen in primary care settings. There is currently insufficient evidence to recommend home versus office-based induction.[12,13,14]

Acknowledging the dire need for increased access to effective treatment for OUD, patients and providers are eager to better understand if home or office-based induction in the primary care setting leads to more successful short-term stabilization and long-term maintenance treatment and patient outcomes. They also question whether certain patient characteristics, such as substance use history, executive function, and social determinants of health, are associated with better long-term outcomes in patients receiving one method versus the other.

III. Preliminary Studies/Progress Report:

Medication assisted treatment (MAT) is a proven, guideline concordant strategy to treat OUD.[10,11] Historically, MAT has often involved receiving daily doses of methadone at a certified addiction treatment center (methadone clinic). However, the primarily urban locations of these centers and treatment protocols present substantial barriers to access to care. Office-based opioid treatment (OBOT), such as MAT with buprenorphine in primary care clinics, is a proven strategy to treat OUD and is slowly becoming accessible to patients through primary care.[5,6,7,8] MAT provided by primary care practices has the potential to greatly increase access to care, reduce preventable deaths from overdose, and improve quality of life.[15]

Treatment with buprenorphine begins with "induction," during which patients discontinue their opioids, begin withdrawal, and receive the first one to four doses of buprenorphine. Induction may be performed using either an office-based or home induction protocol. Both strategies are evidence-based and considered standard care.[10,11,12] However, previous research has focused on short-term outcomes. No data are available indicating which method leads to superior long-term maintenance treatment outcomes or which option might be a better fit for certain types of patients.

MAT consists of 3 phases: induction, stabilization, and maintenance. For induction, patients discontinue their opioids, begin withdrawal, and receive the first one to four doses of buprenorphine. The neurobiology of addiction requires that a person achieve a level of withdrawal in order for buprenorphine to be effective, which requires patients take their last

opioids 24 – 72 hours prior to induction. If given too early, buprenorphine can rapidly precipitate an intense opioid withdrawal. Because of concerns about this risk, inductions have often occurred in the primary care clinic with monitoring from the clinic staff ("observed" or office-based induction). Timing the last use of opioids such that patients arrive during available office hours for induction while in withdrawal is challenging. Further, office induction is a resource intensive activity requiring space, provider and staff time, and patient effort. Based on our previous work (IT MATTTRs), office-based induction might require one to three hours of patient time in the office, two to four staff interactions, and one to three prescriber interactions. Other care providers, including behavior health, peer counselors, and ancillary staff may have additional interactions with the patient. Busy primary care practices, rural health centers, Federally Qualified Health Centers, and other safety net clinics must balance access to onsite care for many patients with the needs of an individual patient with OUD. Some practices are reluctant to dedicate this amount of resource for one patient encounter.[16,17] Although resource intense, these same office-based resources might also create a positive cumulative benefit for patients not available in-home inductions, including enhanced patient-provider-practice interaction and support for patient behavioral factors (motivation, planning, and action control) known to affect long-term adherence to treatment.[18,19,20,21]

Home (or "unobserved") induction is an alternative strategy that poses fewer practice challenges and allows patients to begin buprenorphine therapy according to their schedule. For home induction, patients can stop opioids at any time and start buprenorphine when they reach the appropriate level of withdrawal. Stigma associated with OUD and difficulty with transportation may present other barriers to starting MAT at the clinic. Some patients may prefer undergoing MAT induction in the relative comfort of their own home. However, while potentially more convenient, some patients lack social supports and relationships[22,23,24] and the cognitive ability to set goals and follow through on plans (low executive function) often necessary for successful MAT induction and treatment.[25,26,27]

Patients typically transition into the stabilization phase of MAT 24 to 48 hours after induction. Stabilization may last one to three months. During this time, patient motivation is challenged, temptations and roadblocks may jeopardize plans made by the patient (internal plans) and plans made by the practice and family (external plans), and actions of treatment maintenance are not yet routine. Successful induction may provide a firm foundation for this difficult stabilization phase. As patients begin routine medication use, they no longer suffer withdrawal symptoms and do not engage in opioid-seeking behavior. MAT initially focuses treatment on the opioid dependence. As patients successfully maneuver through MAT stabilization, they are often able to address the behavioral factors associated with their addiction. At this point, patients enter the maintenance phase of MAT, during which they are more able to turn their attention to other elements of their life. They can address many of the social determinants of their health, such as education and employment, along with mental, emotional, and behavioral health issues. Successful induction sets the stage for successful MAT stabilization and long-term maintenance treatment.

Important differences between the activities that occur during a home versus office-based induction may influence the induction success, short-term stabilization, long-term maintenance treatment, and quality of life outcomes. A recent review identified ten studies describing home induction, with just one randomized controlled trial with 20 patients observed for up to 12 weeks.[12,14] However, no large-scale, multi-center, randomized comparative effectiveness research has compared home versus office-based induction on long-term outcomes for patients

suffering from OUD receiving treatment in primary care settings. Additionally, no studies have explored whether specific types of patients are more likely to benefit from home versus office-based induction. There is currently insufficient evidence to recommend home versus office induction.[12,13,14]

Providers and patients seek clarity about the benefits and risks of home inductions compared to office-based inductions. This comparative effectiveness study of the existing, evidence-based home induction and office-based induction strategies for MAT will examine long-term outcomes to help fill this critical evidence gap.

IV. Research Methods

A. Outcome Measure(s):

HOMER is a randomized comparative effectiveness research study to compare differences in short-term and long-term outcomes in patients who begin medication assisted treatment (MAT) for opioid dependence or opioid use disorder (OUD) with an unobserved induction (home induction) or an observed (office-based) induction. The primary outcomes are:

1. Number of days of buprenorphine treatment between day one and day 270 of treatment
2. Number of days of use of illicit opioids between day one and day 270 of treatment.
3. Percent of patients in each group who took buprenorphine on ≥80% of the days between day one and day 270.
4. Percent of patients in each group using illicit opioids on <10% days from induction to nine-month follow-up.
5. Quality of life and social functioning

B. Description of Population to be Enrolled:

The populations to be enrolled will live in the United States, including urban and rural regions. Human subjects participation will include both patients and practice staff and clinicians.

- Participating patients age 16 and older identified by their clinician at a participating primary care practice as having opioid dependence or OUD and agreed to receive MAT. Participants will be patients of diverse types of practices, including Federally Qualified Health Centers, Rural Health Clinics, private practices, community health centers, and hospital-based practices.

  Exclusion criteria: Patients will be excluded if they are hypersensitive to buprenorphine or naloxone, known to have serum aspartate aminotransferase or alanine aminotransferase levels greater than five times normal, and/or diagnosed with severe, untreated psychiatric illness. Patients with an existing preference for office-based or home MAT induction will be not be eligible. If a patient is deemed ineligible for study participation or does not wish to participate, induction for MAT will occur using the practice's usual clinical protocol.

- Clinicians and staff at participating primary care practices. Primary care practices with at least one waivered prescriber currently treating patients with OUD with buprenorphine will be invited to participate.

C. Study Design and Research Methods

The study is a randomized, parallel group, two-arm pragmatic comparative effectiveness trial of two standard care models for induction (home or office-based) for MAT in primary care practices. Randomization will be at the patient level. We have designed our study to measure outcomes based on the primary comparators and important factors that may influence both the short-term and long-term success of MAT.

Community and Patient Engagement

This proposal is the direct result of questions and comments regarding office-based versus home inductions for MAT from practices, patients, community members, stakeholders, and practice facilitators around the country involved with the IT MATTTRs™ Program. Local community and patient advisory councils from the HPRN and CaReNet contributed to the development of this study and are dedicated to ongoing collaboration in all aspects of the study. We will draw from our existing patients and community councils to form a Project Community Advisory Council, adding a small number of new members to round out the types of expertise and experiences present. We will hold meetings and regularly communicate with the Council between meetings to maintain progress and general communication. Specific activities will include the development of practice and patient recruitment materials, reviewing and assisting in the selection of survey instruments and study protocols, interpreting results, preparing and giving presentations at local and national meetings, and identifying and participating in other dissemination activities. Community members have and will participate in the Human Subjects Training for Community Members (submitted to COMIRB 7/7/2020, resulting from meeting with Alison Lakin, PhD; Warren Capell, MD; Marc Ringel, MD (CCTSI); Jack Westfall, MD; and Linda Zittleman, MSPH; 7/15/2014).

Recruitment

Practice Recruitment

Primary care practices with at least one waivered prescriber currently treating patients with OUD with buprenorphine will be invited to participate. The study goal is to recruit 100 practices nationwide. In Colorado, we will recruit practices from the State Networks of Colorado Ambulatory Practices and Partners (SNOCAP) and will partner with other organizations that work with primary care practices to notify practices of the study. Based in the Department of Family Medicine, SNOCAP is the overarching name for the collaborative affiliation of practice-based research networks in Colorado that joined together to improve communication, facilitate joint research projects, and share research staff and resources. The SNOCAP PBRNs assisting with this study work closely with diverse primary care practices in urban and rural regions across the state.

Nationally, the Academy of Family Physicians (AAFP) National Research Network will recruit practices. The AAFP is the national professional association of family physicians. The AAFP NRN has conducted more than 80 studies and has over 2,300 clinician members in 1,000 diverse practices covering all 50 states, the District of Columbia, and Puerto Rico.

Practices will be notified of the study via recruitment flyer. The flyer will be sent to practices via emails and shared at in-person visits. The practice recruitment flyer will be submitted to COMIRB for approval prior to use.

Participant Recruitment

- Patients

This study will enroll patients that the primary care provider believes would benefit from MAT with buprenorphine. As this study will occur with the patient's health care medical provider, the recruitment for participation in this study will occur in accordance with guideline-concordant screening, as part of usual care, by the practice care team to identify patients with OUD and patients with chronic pain that have opioid dependence and could benefit from MAT with buprenorphine. Primarily these are patients with opioid dependence and either 1) addiction as defined by DSM-V criteria for OUD and/or 2) chronic pain with long-term, high dose opioid use (greater than one year and morphine equivalent daily dose higher than recommended by the CDC). Participating practices will describe the options for induction (office or home) to patients who are opting to receive MAT, which is part of their usual care. Patients who are willing to use either type and meet criteria will be informed of the study verbally and given a study information sheet. If the patient is interested and willing to learn more about the study at that visit, referred here to as Time 1, the practice will call the central study team staff, who will describe the study in detail to the patient, including that participation involves randomization to office-based or home induction. If the patient is interested in participating, the study team staff will confirm eligibility, consent the patient, randomize the patient to the *office-based induction* arm or the *home induction* arm, and contact the practice with the study arm.

This study aims to enroll 1200 participants to reach a goal of 1000 retained participants receiving MAT (allowing for attrition prior to MAT induction). In Colorado, enrollment goals are 660 to retain 550 receiving MAT. We anticipate an average of 10 patients per practice will be screened for OUD or identified as opioid dependent, accept MAT induction, enroll in the study (accepting randomizing into home or office-based induction), and have monitoring data entered into the Practice Participant Tracker.

- Practice Clinicians and Staff

Participating clinicians and staff will be invited to participate in key informant interviews (approximately 2-3 people per practice) and surveys (all practice members). Participants will be identified from practice rosters maintained by the partnering PBRNs and with assistance from practice managers to confirm the number of practice staff.

Research Methods

<u>Conceptual Framework</u>

The project evaluation will be informed by the conceptual framework for this study, which is based on the Rubicon model of action phases (Achtziger & Gollwitzer) and self-determination theory (Ryan & Deci). The Rubicon model is a model of behavior change that addresses motivation to change and turning motivation into action. Phase 1 focuses on motivation (deciding to change and setting goals). Phase 2 focuses on planning (determining how, where, when and for how long a goal will be pursued,). In Phase 3, the focus is action

control (exerting effort to execute the plan). Phase 4 focuses on evaluation (consideration of the value of continued goal pursuit).

Randomization

Patients who meet eligibility criteria and consent to participate will be randomized to either the *office-based induction* arm or the *home induction* arm. Both induction methods are evidence-based approaches for initiating MAT in patients with OUD.[9,10,11] Participating practices will receive a suggested protocol for both office-based and home inductions. However, practices and providers experienced in MAT may have their own existing protocols for office and home induction. Given the pragmatic nature of this trial, we will allow some variation in induction protocols.

Randomization will occur at the patient level. Patients will be informed that participation includes randomization by the initial information handout they receive from the practice and again by the research team member during the consent process. Randomization will occur immediately following consent. A study coordinator will inform the participant of their randomization assignment. As consent will be completed by phone with the patient, the study team member will inform the participant of the study arm assignment (randomization result) while on the phone with the participant and then immediately inform the practice of the patient's assigned induction type.

A block randomization process will be used where patient participant "slots" will be randomized for each practice in advance of recruitment to ensure equal distribution between arms at each practice. This will increase our power to detect differences between study arms, as opposed to a cluster randomized trial. Further, this use of block randomization will provide the best opportunity for each practice to enroll patients in either arm of the study and for regular monitoring of enrollment across arms. Few previous studies on home and office-based MAT induction have been randomized trials. The study findings will be the result of rigorous research design and methods.

Office-based and Home Induction Protocols

Standard observed, office-based induction includes the practice staff or clinician assessing the patient for level of withdrawal, administering the first dose of medication, monitoring the patient, and administering subsequent doses of medication at the practice. Patients are given a follow-up plan, including a dosing schedule for the next day, indications of when to call the practice, a follow-up contact schedule (calls or in-person appointment the next day), and an appointment for later in the week.

Standard unobserved, home induction includes giving patients instructions on induction steps. Participants doing home inductions self-assess and determine when they are in adequate withdrawal to administer their first dose of buprenorphine. With home induction, patients administer their initial dose of buprenorphine, assess withdrawal symptoms using the SOWS, and administer subsequent doses of buprenorphine themselves. If practice is aware of when home induction will occur, a member of the practice team might contact patient to assess progress as needed. Patients follow up as agreed upon with their practice team.

Consent

Subjects recruited for this study include practice staff and patients. This project poses minimal risk to study participants. Participation is voluntary. Patient participants may be

uncomfortable answering questions about buprenorphine prescription or illicit opioid use and other aspects of their lives. Participants can refuse to answer any questions they want. Key informant interview participants can determine the information they share during their interviews. Consent will be obtained..

Patients

Interested potential participants (with a diagnosis of OUD and seeking to begin MAT) will receive a study informational packet at their clinic, which will include a copy of the Postcard Consent Form. The practice will call the research team member assigned to them with the patient's contact information for patients that have expressed interest in the study and given the practice permission to initiate contact with the research team. If the patient is interested and willing to learn more at that visit, referred here to as Time 1, the practice will call the research team member assigned to them with the patient's contact information for patients that have expressed interest in the study and given the practice permission to initiate contact with the research team. A member of the research team will describe the study in detail to the patient, confirm eligibility, consent and randomize the patient, and contact the practice with the study arm. If this step cannot occur at Time 1, the practice will transmit to the central study team the interested and eligible patients' contact information for later informed consent and randomization. The goal will be to contact eligible, interested patients within 4 days of Time 1. At what is referred to here as Time 2, the patient will undergo MAT induction, per protocol for the method (home or office-based). We will strive to complete consent and randomization such that practices schedule Time 2 (induction) with 5 – 15 days of Time 1.

A signed, hard copy of the Postcard Consent Form will not be required from participants. The research team member will obtain and document verbal consent in REDCap. The Postcard Consent Form will explain that participation is voluntary and that they can discontinue participation at any time, the nature of participation, and that all data are confidential. For children age 16 and 17, we will obtain parent or guardian contact information to contact the parent. Parents or guardians will be informed of the study purposes and procedures. We will contact the child to obtain assent only after consent is obtained from the parent or guardian.

Although patients will be consented, each survey will still include a brief Information Sheet reviewing the conditions of participation, including that participation is voluntary and confidentiality. In addition to documentation of verbal consent by the study team, completion of surveys and assessments will imply consent.

Practice Clinicians and Staff

Practice team members' survey will include an Information Sheet. Completion of surveys and interviews will imply consent.

For practice team members participating in key informant interviews, the research team interviewers will review the content of an Information Sheet with interview participants. All efforts will be made to provide an environment where questions are welcome and where questions can be answered privately, if preferred by the participant.

All members of the project team are trained in human subjects research and HIPAA.

Timeline

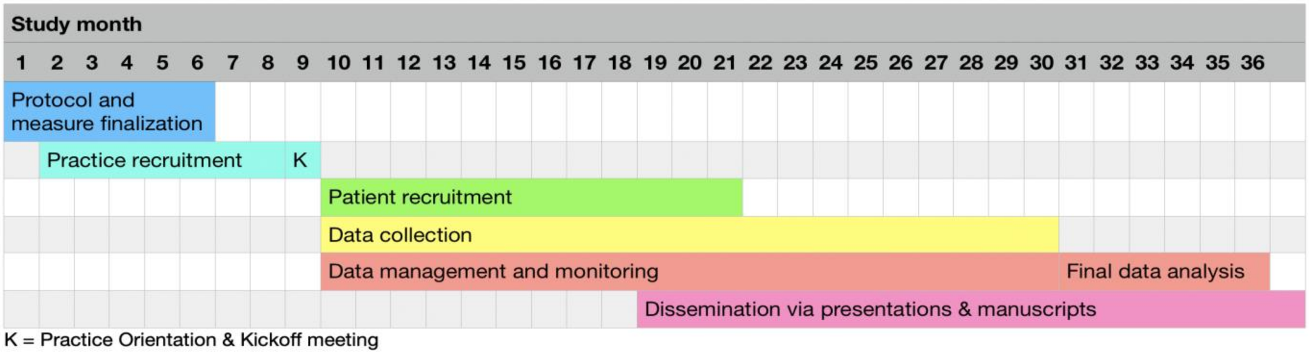

<u>Sustainability and Dissemination</u>

Dissemination of research findings requires commitment to both peer reviewed presentation and publication and to strategic efforts with patient and community partners and key stakeholders. Throughout this study, we will distil lessons learned about home and office-based MAT induction to support the provision of MAT in diverse primary care settings. We will utilize formal and informal networks and relationships to disseminate progress and findings.

The research team will identify opportunities for sharing findings throughout the life of the grant to local providers, practices, and communities. We will work closely with the University of Colorado and the American Academy of Family Physicians (AAFP) media departments. We will share findings through regular state and national communication and peer-reviewed publications. We propose regular newsletter articles for the participating practice-based research networks beginning in the first three to six months of the grant. This will provide knowledge of the planned activities and stimulate interest in the project. We will collaborate with local media outlets, including newspapers, television, and radio, to provide updated information on planned efforts, progress, and final outcomes.

D. Description, Risks and Justification of Procedures and Data Collection Tools:

<u>Practice Data</u>

This study will include a process evaluation to describe factors that provide important practice contextual information relevant to data interpretation.

MAT Implementation Checklist

Upon recruitment, we will administer a MAT Implementation Checklist one person in the practice, such as a Practice Manager, to establish practices' baseline MAT protocols (including any planned adaptations). Where variations exist, we will ensure they do not impact feasibility of data collection and patient safety. If they do not impact these, we will document the variations and determine how they will be included in the outcomes analysis. A study team member will conduct regular monthly check-ins with each practice to establish fidelity to each practice's MAT protocol and document adaptations, review patient recruitment progress, obtain patient drop-out information, and assess utilization of the Practice Participant Tracker (see below). The Checklist will require 8-10 minutes to complete. Practices will have the option to complete the form online or mailed a hard copy (along with a postage-paid envelope to mail completed

Checklists back to the research team at the University of Colorado). The MAT Implementation Checklist is submitted for review and approval (8/24/2020).

Key Informant Interviews

We will conduct semi-structured key informant interviews with approximately 80 individuals from 30-40 participating practices (stratified based on the final sample characteristics and protocol variation) as they complete their follow-up phase (months 18 – 30 of the study). Interviews will be conducted with 2-3 people (combination of clinicians, nurse, and support team members) at each site. The interviews will review MAT protocols and document any adaptions, reasons for the adaptations, unexpected changes and to assess frequency of patient follow-up. These interviews will capture additional contextual information about how the experience differs for home versus office-based inductions.[28] Participants will receive a $50 gift card.

Interviews will be conducted by research team members by phone and will last approximately 30 minutes. Interviews will be recorded, with respondent's permission. Interviews will be transcribed. No PHI (protected health information) will be collected. No names will be attached to the records. Data will be de-identified. Data from transcripts will be entered into Atlas.ti (qualitative data analysis software) by a project team member for analysis and will be anonymous. Audio files and data from transcripts will be stored on a secure server maintained by the Dept. of Family Medicine (DFM) IT team. Only the study team will have access to the data. Any hard copy notes will be locked in a secure, locked office in DFM. Results will be reported in aggregate format. After analysis is complete, data will be stored according to NIH guidelines. An Information Sheet and Interview Guide will be submitted to COMIRB for approval prior to use.

Surveys

Practice team members will be asked to complete a short Knowledge Survey to measure participants' knowledge of and attitudes towards OUD, MAT, and their practices' OUD management and MAT delivery. They will also be asked to complete a Practice Culture Assessment to help describe practice team's perception of practice characteristics that influence its decision-making processes, leadership, and other aspects of the practice culture. The Practice Knowledge Survey and Practice Culture Assessment are submitted for review and approval (8/24/2020).

Respondents will have the option the complete their surveys online using REDCap or on hard copy. With hard copies, participants will be given a postage-paid envelope to mail completed surveys back to the research team at the University of Colorado.

No names will appear on the surveys. Surveys pose minimal risk to practice members. There is a minimal risk of loss of confidentiality. Survey data from will be confidential and only used by the study team.

<u>Patient Data</u>

Practice Tracker

Patient-level data will include the use a Practice Participant Tracker by participating practices to document participant information and clinical care activities. The Tracker will be a binder organized with numbered tabs for each participating patient or electronic Excel file. A spreadsheet for each patient will include a virtual 300-day timeline from initial identification through final data collection. This Tracker will be used to record patient enrollment, completion

of patient MAT education, induction type, induction Day 1 date, prescriptions and refills, urine buprenorphine and urine opioid test results, contacts with patients (when and whether these be via phone, e-mail, or in person), and continuation or discontinuation of treatment. Practices may record information either on paper or electronically with periodic transmission to the central data management team electronically or via secure fax. Summary notes will be recorded from the monthly check-in calls with practices. A template copy of the Practice Participant Tracker will be submitted to COMIRB for review and approval prior to use. Practices will receive $200 for each patient enrolled from their practice (up to 12) and $35 per patient per month for up to nine months of monitoring maintaining follow-up with each patient in their Practice Participant Trackers, paid at the end of the data collection phase.

Surveys

Patient participants will be asked to complete questionnaires and assessments (surveys) pertaining to social determine of health and quality of life. Surveys will be administered at baseline, 30 days, 90 days, and 270 days. Participants will receive a $50 gift card (e.g., Amazon, Walmart) per time (baseline, 30 days, 90 days, and 279 days), totaling up to $200 in gift cards. Respondents will have the option the complete their surveys online using REDCap or on hard copy. With hard copies, packets will include a postage-paid envelope to mail completed surveys back to the research team at the University of Colorado. Final selection of these questionnaires and assessment will be made and submitted to COMIRB for review and approval prior to use.

A summary of the study measures and potential data collection tools is shown in Table 1.

Table 1 Outcomes, Measures, and Potential Data Collection Tools

Outcome	Measure	Data Collection Tool(s)
**Primary Outcomes**	*Timing of data collection indicated by *, **, ✦, ¥, ◆*	
Number of days patient took buprenorphine between Day 1 and Day 270✦	▪ Prescription refills✦<br>▪ Patient self-report of number of days buprenorphine taken✦	▪ Practice Participant Tracker
Number of days patient took illicit opioids between day 1 and day 270[**,¥]	▪ Patient self-report of number of days other opioids taken✦<br>▪ Patient urine opioid test results[¥]	▪ Practice Participant Tracker
Took buprenorphine on >80% of days between 1 and 270[52,53]	▪ Initial prescription and refills✦<br>▪ Patient self-report of number of days buprenorphine taken✦<br>▪ Patient urine buprenorphine test results[¥]<br>(Above data converted to yes/no measure) [52,53]	▪ Practice Participant Tracker
Percent (%) of patients that took other opioids on <10% of days between 1 and 270	▪ Patient self-report of number of days other opioids taken✦<br>▪ Patient urine opioid test results[¥]	▪ Practice Participant Tracker
**Patient-Centered Outcomes±**		
Social Determinants of Health and Quality of Life	Patient surveys completed per clinical care on quality of life (e.g., symptoms, side effects, depression), social connectedness, loneliness, and other select determinants of health**	▪ NIH PROMIS or SF 12 or 20 [56,57]<br>▪ Activity and Participation Questionnaire[60]<br>▪ Social & Community Opportunities Profile[61]<br>▪ UCLA 3-item Loneliness Scale[62]<br>▪ American Academy of Family Physicians Social Determinants of Health tool[63]
**Patient-Level Moderators and Mediators±**		
Moderators<br>▪ Substance Use History<br>▪ Substance Use Appeal<br>▪ Executive Function	Patient Surveys completed per clinical care on quality of life*	▪ PROMIS short form measures – Appeal and Severity of Substance Use[56]<br>▪ Behavior Rating Inventory of Executive Function–Adult, self-report version) [27]

Mediators<br>▪ Motivation<br>▪ Planning<br>▪ Automaticity	Patient Surveys completed per clinical care on quality of life**	▪ Health care climate questionnaire<br>▪ Intention measure[64]<br>▪ Planning measure[64]<br>▪ Self-Report Habit Index[65]
Demographics	Patient Surveys*	▪ Patient Demographic Survey
**Practice-Level Moderators**		
Contextual Factors	▪ Aspects of practice culture, leadership, decision-making collected from all members of participating practices*<br>▪ Practice type, number of providers, number of waivered providers, practice MAT training, duration of MAT provision completed by knowledgeable practice member*	▪ Practice Culture Assessment<br>▪ Practice Characteristics Survey
Process Measures	▪ Medication assisted treatment protocols, completed by knowledgeable practice member*<br>▪ Fidelity & adaptations to MAT protocols◆	▪ IT MATTTRs MAT Implementation Checklist[29]
*Collected at baseline only (patient data collected during consent process)<br>**Collected at baseline, 30 days, 90 days, and 270 days after induction<br>✦ Collected at 30, 90, and 270 days after induction<br>¥ Collected at ≥2 office visits<br>◆Collected during Monthly Check-In Calls<br>± To be discussed with the Project Community Advisory Council to determine final selection of instruments and measures		

Contact Information

Contact information for patient participants, including name, address, phone numbers and email address, will be obtained in order to remind participants to complete scheduled surveys and to send participants their gift cards each time they complete surveys and assessments. Alternate contact information will be obtained for two other people, family members not living with the participant or close contacts, who may be knowledgeable about the participant in the event that the participant cannot be contacted for subsequent longitudinal follow-up. Alternate contact information will include name, address, phone numbers and email addresses. All contact information will be stored securely in a database separate from that developed for the clinical and survey data and accessible only to the research team responsible for tracking patient participant payments (gift cards).

Tracking Procedures

No names will appear on the surveys or Practice Participant Tracker data. Practice member surveys will include a unique ID linked to the practice only. Each patient participant will be assigned a unique ID by the research team. The unique ID keys will be stored separately from research data. We will use REDCap to send unique survey links to participants completing their surveys online. Hard copies of surveys will include the appropriate unique ID (practice-level for practice members and patient-level for participating patients). Survey data from written surveys will be entered into REDCap by a project team member. Hard copies will be locked in a secure filing cabinet in a locked office space in the Department of Family Medicine.

The unique patient participant ID will be used by practices to transmit de-identified data from the Practice Participant Tracker to the study team.

This project poses minimal risk to study participants. Participation is voluntary. Patient participants may be uncomfortable answering questions about buprenorphine prescription or illicit opioid use and other aspects of their lives. Participants can refuse to answer any questions

they want. Consent will be required. Key informant interview participants can determine the information they share during their interviews.

E. Potential Scientific Problems:

This is a comparative effectiveness research study, randomized at the patient level, to compare short-term stabilization and long-term maintenance treatment outcomes of home MAT induction to office MAT induction for patients suffering from OUD and opioid dependence.

F. Data Analysis Plan:

Sample Size and Power:

We aim to recruit 100 practices, enroll 1200 patients to reach a goal of 1000 patients receiving MAT induction (allowing for attrition prior to MAT induction), and randomize patients evenly to each study arm. For some outcomes (e.g., quality of life), we allow for ~20% attrition (leaving 800 patients total, 400 per arm). Some outcomes will not be affected by patient dropout, so we give a range of sample size estimates. With patient-level randomization, the impact of clustering will be minimal and is not considered here. For the dichotomous outcome of treatment retention, a sample size of 500 patients per arm will provide 81% power to detect a 9% difference in long-term treatment between study arms with alpha=.05 (e.g., 45% vs 54%). For continuous outcomes (e.g., quality of life, % of days taking buprenorphine out of 270 days), a sample size of 400 to 500 patients per arm will provide 81% power to detect an .18 to .20 SD difference (small) between study arms with alpha=.05.[30]

Randomization to Condition

Patients will be randomized to either the office-based MAT induction arm or the home MAT induction arm. An average of 10 patients per practice will be enrolled, with an allowable range of 6 – 20 patients. Block randomization will be used to ensure an equal number of patients in each arm. The "slots" will be randomized for each practice in advance of recruitment to ensure equal distribution between arms at each practice. The result will be approximately 500 patients per study arm. For some outcomes (e.g., quality of life), we allow for ~20% attrition (leaving 800 patients total, 400 per arm).

Quantitative Analytic Plan

After generating initial descriptive statistics on patient and practice characteristics, we will report on differences between: (1) intervention arms and (2) patient dropouts vs. non-dropouts. We will use an intent to treat analysis to assess the effect of the interventions on long-term treatment. We will examine baseline characteristics of patients in both arms using Pearson's $\chi 2$ tests for categorical variables and t-tests for continuous variables to assess for unbalanced confounders. Patient engagement in treatment at 30, 90, and 270 days will be analyzed. Given that the comparators employ different strategies that might affect long-term treatment outcomes, we hypothesize that we will observe a difference in outcomes between the two induction strategies. Patient-level covariates will be screened in bivariate analyses and included in multivariate analysis if related to the outcome at $p<.2$ or are associated with dropout.[31] Covariates (to adjust for potential confounding) and potential moderators will include age, gender, race/ethnicity, comorbidities, and other sociodemographic and health-related variables.

In the event normality assumptions are not met, we will use transformations to normalize distributions, ordinal or Poisson regression where appropriate, and/or the appropriate link function (e.g. logit link for dichotomized measures).[32,33,34] We will employ intent to treat analyses using general (generalized) linear mixed models (GLMMs) to incorporate hierarchical and longitudinal data structures.[33,35,36,37,38,39] Hypothesis tests will be two-sided with α =.05 or p values reported. Goodness of fit statistics and model fitting diagnostics will be used to assess influential points, outliers, overdispersion and heteroscedasticity and to evaluate alternative model specifications. All statistical analyses will be performed using SAS version 9.4 (SAS Institute Inc., Cary, N.C.).

We will use general (or generalized) linear mixed models, adjusted for covariates for analysis of primary and secondary outcomes. Even though randomization will be at the patient level, patients are still clustered within practices. Therefore, we will include practice random effects as well as patient random effects (for longitudinal data). Likelihood based models using all available data are the preferred method for analyzing longitudinal data with dropout under MAR conditions.[40,41,42] This will be our primary analysis where dropout affects repeated measures over time.

Specific Tests and Analysis Plans for Outcomes, by Hypotheses

H1: Patients using observed (office-based) MAT induction will have a greater number of days of buprenorphine treatment than patients using unobserved (home) MAT induction at nine-month follow-up.

The outcomes for these analyses will be the total number of days of buprenorphine treatment during the nine-month follow-up period. The structure of the data is hierarchical (patients nested within practices). Intervention is an indicator variable: 0= home-based induction, 1= office-based induction. Covariates will be included in all models but are not shown here for ease of understanding. Since we anticipate the distribution will be over-dispersed, we will not use a Poisson model. Instead, we will first examine the distribution of the outcome data (counts and/or proportions) and explore alternatives such as negative binomial (or possibly beta binomial on proportions), using model diagnostics and goodness of fit measures to select the best approach. For the negative binomial model, modeling counts (Y_{ij}) we have

$$\text{Log}\ (Y_{ij}) = \gamma_{00} + \gamma_{01}\,(\text{intervention}) + \gamma_{02}(X_{i,}) + u_{0j}$$

where γ_{00} represents the mean (in terms of logs) for home-based induction; γ_{01} represents the difference between means for the office-based vs home induction group; γ_{02} is the effect of patient level covariates on log counts, and U_{00j} is a practice random effect. Thus, the intervention effect of office-based vs home induction can be tested as H_0: $_{101}$=0 vs H_1: $\gamma_{01} \neq 0$.

H2: Patients using office-based MAT induction will be more likely to take buprenorphine on ≥80% of the days between day 1 and day 270 than patients using home MAT induction.

The outcome for this analysis will be buprenorphine use on ≥80% of days (yes/no). We will use mixed effects logistic regression models (generalized linear mixed models with logit link) to analyze the data. The model will be similar to that shown above, with a random effect for practice and patient level covariates, along with a fixed effect for intervention group.

H3: Patients using office-based MAT induction will report greater improvement in quality of life, social determinants of health and social functioning over a nine-month follow-up period than patients using home induction.

Outcomes for this analysis will be scores on quality of life, social functioning, social determinants of health assessments at baseline, 30 days, 90 days, and 270 days. We will use random coefficient growth curve models to determine whether trajectories on quality of life and other patient reported measures for patients receiving home induction compared to patients receiving office-based induction (time x arm interaction term in mixed model notation).

H1a, 2a, 3a: Intervention effects on primary outcomes (medication-taking and quality of life) will differ for patients in subpopulations of interest, defined by high executive function, shorter history of substance use, and lower substance use.

We will conduct exploratory analyses to test for potential effect modification (moderator of intervention effectiveness) by selected patient characteristics. The general hypothesis for these moderator analyses is as follows: The effects of the home versus office-based induction on patient medication-taking (and other primary outcomes) will differ by baseline levels of executive function and substance use (amount and shorter history), dichotomized by baseline scores.

For hypotheses 1 and 2, moderator analyses involve inclusion of main effect for the moderator and a moderator x intervention term. The interaction term tests the differential effect of the intervention on the outcome for subgroups defined by the moderator variable. For hypothesis 3, moderator analyses involve inclusion of the main effects for time, arm, moderator variable, time x arm, time x moderator, arm x moderator, and time x arm x moderator interaction term in the model. The three-way interaction term tests for differential intervention effectiveness by subgroups identified by the moderator variable. Due to the exploratory nature of these analyses, we do not plan to adjust for multiple comparisons in moderator analyses using the subgroups defined above. However, interpretation of results will include reporting on all subgroup analyses and take into account the number of subgroup analyses performed.

Qualitative Data Analysis:

Qualitative data from interviews will be recorded, transcribed, and placed into the qualitative software program atlas.ti for analysis. Data will be reviewed for themes and lessons learned. Quotes from the key informant interviews may be used to illustrate a theme but will not be identified. Analysis will be in the form of truth tables. The interview guide will be submitted for COMIRB review when ready, prior to use.

G. Summarize Knowledge to be Gained:

This study will enable us to assess the impact of two different approaches to MAT induction on long-term treatment engagement and patient-centered outcomes and to determine if certain patient characteristics are associated with more successful treatment engagement based on induction method. With opioid use and drug-related deaths increasing, study findings will be critical and timely. Knowledge gained will guide ongoing efforts to help patients and providers make informed decisions related to MAT expand MAT in primary care settings, improve patient experience, and reduce morbidity and mortality due to opioid dependence and OUD.

H. References:

[1] Bonnie RJ, Kesselheim AS, Clark DJ. Both urgency and balance needed in addressing opioid epidemic: A report from the National Academies of Sciences, Engineering, and Medicine. JAMA. 2017;318(5):423-424.

[2] Rosenblatt RA, Andrilla CHA, Catlin M, et. al. Geographic and specialty distribution of US physicians trained to treat opioid use disorder. Ann Fam Med. 2015;13(1):23-26.

[3] Kolodny A, Courtwright DT, Hwang CS, et. al. The prescription opioid and heroin crisis: A public health approach to an epidemic of addiction. Annual Review of Public Health. 2015;36:559-574.

[4] Saitz R, Daaleman TP. Now is the time to address substance use disorders in primary care. Ann Fam Med. 2017;15(4):306-308.

[5] Johnson R, Chutuape M, Strain EC, et al. A Comparison of Levomethadyl Acetate, Buprnorphine, and Methadone for Opioid Dependence. N Engl J Med. 2000;343(18):1290-7.

[6] Fudala PJ, Bridge P, Herbert S. et al. Office-Based Treatment of Opiate Addiction with a Sublingual-Tablet Formulation of Buprenorphine and Naloxone. N Engl J Med. 2003;349:949-958.

[7] Thomas CP, Fullerton CA, Kim M, et. al. Medication assisted treatment with buprenorphine: Assessing the evidence. Psychiatr Serv. 2014;65(2):158-170.

[8] Rosenblatt RA, Andrilla CHA, Catlin M, et. al. Geographic and specialty distribution of US physicians trained to treat opioid use disorder. Ann Fam Med. 2015;13(1):23-26.

[9] Kampman K, Jarvis M. American Society of Addiction Medicine National Practice Guideline for the Use of Medications in the Treatment of Addiction Involving Opioid Use. J Addict Med. 2015. p. 358–67.

[10] Cunningham CO, Giovanniello A, X Li, et al. A comparison of buprenorphine induction strategies: patient-centered home-based inductions vs standard-of-care office-based inductions. J Subst Abuse Treat. 2011. Jun;40(4):349-56.

[11] Center for Substance Abuse Treatment. Medications for Opioid Use Disorders. Treatment Improvement Protocol (TIP) series 63. DHHS Publication No. (SMA) 18-5063. Rockville, MD: Substance Abuse and Mental Health Services Administration, 2018.

[12] Lee JD, Vocci F, Fiellin D. Unobserved “Home” Induction Onto Buprenorphine. J Addict Med 2014;8: 299–308.

[13] Lagisetty P, Klasa K, Bush C, et. al. Primary care models for treating opioid use disorders: What actually works? A systematic review. PLoS ONE. 2017;12(10):e0186315. https://doi.org/10.1371/journal.pone.0186315. Accessed 10 February 2018.

[14] Gunderson EW, Wang XQ, Fiellin DA, et al. Unobsersved versus observed office buprenorphine/naloxone induction: a pilot randomized clinical trial. Addict Behav. 2010;35(5):537-40.

[15] Wakeman SE, Barnett ML. Primary Care and the Opioid-Overdose Crisis - Buprenorphine Myths and Realities. N Engl J Med. 2018. Jul 5;379(1):1–4.

[16] Andrilla CHA, Coultbard C, Larson EH. Barriers rural physicians face prescribing buprenorphine for opiate use disorder. Ann Fam Med. 2017;15:359-362.

[17] DeFlavio JR, Rolin SA, Nordstrom BR, et. al. Analysis of barriers to adoption of buprenorphine maintenance therapy by family physicians. Rural and Remote Health (Online). 2015;15:3019.

[18] Zeldman A, Ryan RM, Fiscella K. Motivation, autonomy support, and entity beliefs: Their role in methadone maintenance treatment. Journal of Social and Clinical Psychology. 2004;23(5):675-696.

[19] Hagger MS, Luszczynska A. Implementation intention and action planning interventions in health contexts: State of the research and proposals for the way forward. Applied Psychology: Health and Well-Being. 2014;6(1): 1-47.

[20] Sniehotta FF, Schwarzer R, Scholz U, et al. Action planning and coping planning for long-term lifestyle change: theory and assessment. European Journal of Social Psychology. 2005;35(4):565-576.
[21] Horne R, Clatworthy J. Adherence to advice and treatment. Health psychology. 2nd ed. Chichester: Blackwell Publishing Ltd. 2010, p 175-88.
[22] Danner UN, Aarts H, de Vries NK. Habit vs. intention in the prediction of future behaviour: The role of frequency, context stability and mental accessibility of past behaviour. British Journal of Social Psychology. 2008;47(2):245-265.
[23] Sheeran P, Webb TL. The intention–behavior gap. Social and Personality Psychology Compass. 2016;10(9):503-518.
[24] Webb TL, Sniehotta FF, Michie S. Using theories of behaviour change to inform interventions for addictive behaviours. Addiction. 2010;105(11):1879-1892.
[25] Mullan B, Wong C, Allom V, et al. The role of executive function in bridging the intention-behaviour gap for binge-drinking in university students. Addictive behaviors. 2011;36(10):1023-1026.
[26] Verdejo-Garcia A. Cognitive training for substance use disorders: Neuroscientific mechanisms. Neuroscience & Biobehavioral Reviews. 2016;68:270-281.
[27] Hagen E, Erga AH, Hagen KP, Nesvåg SM, et al. Assessment of executive function in patients with substance use disorder: A comparison of inventory-and performance-based assessment. Journal of Substance Abuse Treatment. 2016;66:1-8.
[28] Chambers DA, Glasgow RE, Stange KC. The dynamic sustainability framework: addressing the paradox of sustainment amid ongoing change. Implement Sci. 2013;8:117.
[29] MATerials Resource Toolkit. IT MATTTRs Colorado (Implementing Technology and Medication Assisted Treatment and Team Training and Resources Colorado) https://www.asam.org/education/live-online-cme/waiver-training/materials. Accessed 3.28.19.
[30] Cohen, J. (1969) *Statistical Power Analysis for the Behavioral Sciences*. NY: Academic Press.
[31] Wright N, Ivers N, Eldridge S, et all. A review of the use of covariates in cluster randomized trials uncovers marked discrepancies between guidance and practice. J Clin Epidemiol. 2015;68(6):603-609.
[32] Littell R, Milliken GA, Stroup WW, et al, eds. SAS System for Mixed Models. Cary, NC: SAS Institute Inc; 1996.
[33] Hedeker D, Gibbons RD. Longitudinal Data Analysis. Hoboken, New Jersey: John Wiley & Sons; 2006.
[34] Raman R, Hedeker D. A mixed-effects regression model for three-level ordinal response data. Stat Med. 2005;24(21):3331-3345.
[35] Bryk AS, Raudenbush SW, eds. Hierarchical Linear Models: Applications and Data Analysis Methods. Second ed. Newbury Park: Sage Publications; 2000.
[36] Murray D, ed. Design and analysis of group-randomized trials. New York: Oxford University Press; 1998.
[37] Giraudeau B, Ravaud P. Preventing Bias in Cluster Randomised Trials. PLoS medicine. 2009;6(5):e1000065.
[38] Puffer S, Torgerson D, Watson J. Evidence for risk of bias in cluster randomised trials: Review of recent trials published in three general medical journals. BMJ. 2003;327(7418):785-789.
[39] Campbell MK, Elbourne DR, Altman DG. CONSORT statement: extension to cluster randomised trials. Bmj. 2004;328(7441):702-708.
[40] Dempster A, Laird N, Rubin D. Maximum likelihood estimation from incomplete data. Journal of the Royal Statistical Society (B). 1977;39(1):1-38.
[41] Little RJ, Rubin DB. Statistical analysis with missing data. John Wiley & Sons; 2014.
[42] Fairclough DL. Design and analysis of quality of life studies in clinical trials. CRC press; 2010.
